# Maternal adjustment or constraint: differential effects of food availability on maternal deposition of macro‐nutrients, steroids and thyroid hormones in rock pigeon eggs

**DOI:** 10.1002/ece3.1845

**Published:** 2016-01-08

**Authors:** Bin‐Yan Hsu, Cor Dijkstra, Veerle M. Darras, Bonnie de Vries, Ton G.G. Groothuis

**Affiliations:** ^1^ Behavioural BiologyGroningen Institute for Evolutionary Life SciencesUniversity of GroningenGroningenThe Netherlands; ^2^ Comparative EndocrinologySection of Animal Physiology and NeurobiologyKU LeuvenLeuvenBelgium

**Keywords:** Androgens, egg mass, food conditions, maternal effects, thyroid hormones, yolk hormone deposition

## Abstract

In oviparous species like birds, eggs provide the direct environment in which embryos are developing. Mothers may adjust different egg components in different ways in reaction to environmental cues either to adjust offspring development or because of constraints. In this study, we investigated the effects of food quality and quantity before and during egg laying on three different aspects of egg quality: macro‐nutrients (egg and yolk mass), androgens (testosterone and androstenedione), and thyroid hormones (3,5,3′‐triiodothyronine, T3 and l‐thyroxine, T4), using the rock pigeon (*Columba livia*). As expected, egg and yolk mass were significantly reduced for the eggs laid under the poor‐food condition, indicating a maternal trade‐off between offspring and self in allocating important resources. We did not find any significant change in yolk testosterone or their within‐clutch pattern over the laying sequence. This is consistent with the fact that, in contrast with nutrients, these hormones are not costly to produce, but does not support the hypothesis that they play a role in adjusting brood size to food conditions. In contrast, we found that T3 levels were higher in the egg yolks under the poor‐food condition whereas the total T4 content was lower. This change could be related to the fact that iodine, the critical constituent of thyroid hormones, might be a limiting factor in the production of this hormone. Given the knowledge that food restriction usually lead to reduction of circulating T3 levels, our results suggested that avian mothers can independently regulate its concentrations in their eggs from their own circulation. The study demonstrates that environmentally induced maternal effects via the egg can be a result of a combination of constrained resources and unconstrained signals and that thyroid hormones might be an interesting case of both. Therefore, this hormone and the interplay of different maternal effects on the offspring phenotype deserve much more attention.

## Introduction

Over the past 15 years, fuelled by the publication of the book by Mousseau and Fox (1998), a paradigm shift has occurred about the interpretation of maternal effects. Maternal effects are those in which the phenotype of the mother (or father) affects the phenotype of the offspring. Although initially seen as annoying noise for breeding programs, it has now become clear that maternal effects are wide spread among plants and animals, are shaped by and can profoundly affect evolution. Prenatal maternal effects are especially intriguing, as they are often overlooked while the embryo can be especially sensitive for organizing effects of its environment. One important pathway for such early maternal effects is egg quality, both in terms of nutrients (yolk and albumin mass) and regulatory signals (hormones) to which the embryo is exposed in a wide array of animal taxa, ranging from insects to fish, reptiles, and mammals including humans (Bernardo 1996; Groothuis et al. 2005). Although egg mass has received a lot of attention in the past (Bernardo 1996; Krist 2011; Williams 2012), more recently maternal hormones in avian eggs have attracted much attention for several reasons (Groothuis et al. 2005; Gil 2008; von Engelhardt and Groothuis 2011). First, avian egg yolks contain substantial amounts of maternally derived steroid and nonsteroid hormones. Second, their embryo develops outside the mother's body and together with the large egg size this facilitates hormone measurements and manipulation *in ovo* (Groothuis et al. 2005; Groothuis and Schwabl 2008). Third, birds are well‐known models in both behavioral endocrinology and behavioral ecology.

The main hypothesis on the adaptive effect of avian yolk hormones is probably the so called Hatching Asynchrony Adjustment Hypothesis (Schwabl 1993; Groothuis et al. 2005). This is based on the fact that in many bird species females lay several eggs, with an interval of 1–2 days between each egg in a clutch, and that chicks of the last‐laid eggs hatch later with a potential disadvantage in the sibling competition. In the last two decades, many studies in a range of avian species reported that androgen concentrations in egg yolks increase over this laying sequence (reviewed in Groothuis et al. 2005; Gil 2008; von Engelhardt and Groothuis 2011). As there is substantial evidence that yolk androgens enhance embryo development, chick growth and competitiveness (Groothuis et al. 2005; Gil 2008; von Engelhardt and Groothuis 2011), the increasing yolk androgen levels over the laying sequence may serve as a tool for mothers to compensate the drawbacks of hatching asynchrony to the last‐born nestlings within a brood.

However, under poor food conditions it would be advantageous for mothers to adopt a brood reduction strategy. In that case, one would expect female birds to lower the androgen deposition in especially the last laid eggs. Indeed, many studies have shown correlative as well as experimental evidence that levels of yolk hormones, especially androgens, are affected by some internal or external factors, for example, mate attractiveness, social interactions, and the physiological condition of females (reviewed in Groothuis et al. 2005; Gil 2008; von Engelhardt and Groothuis 2011), although the mechanism underlying the hormone accumulation in egg yolks still remains unclear (Groothuis and Schwabl 2008).

Nonetheless, there seems to be no convincing evidence to support the proposition that food availability affects androgen deposition in the egg. To date, only nine studies have experimentally manipulated food conditions before and during egg‐laying to investigate how hormone deposition changes in response, as summarized in Table 1. These studies either applied food supplementation in the field (six studies, Table 1) or fed experimental birds with high‐quality (HQ) or low‐quality (LQ) diet (three studies, Table 1). These studies did not reach consistent conclusions as only two studies found that food‐supplemented females laid eggs containing lower levels of yolk androgens (Table 1). The experimental food effects on within‐clutch variation in yolk androgen deposition are also mixed. Only two of the nine studies detected a significant change of the within‐clutch yolk androgen pattern. In these two studies, one stopped food treatment before egg‐laying (Sandell et al. 2007), i.e. before the time of actual hormone deposition. The other one found that females canaries (*Serinus canaria*) fed with HQ diet laid clutches with a steeper increasing slope of yolk androgens across the laying sequence (Vergauwen et al. 2012). Although these results are consistent with the hypothesis that mothers can flexibly apply yolk androgen deposition to compensate for hatching asynchrony under good food conditions, so far this is the only study providing supporting evidence.

**Table 1:** Previous experimental studies about the effects of food conditions on yolk androgen deposition

Species	Publication	Hormone	Food treatment	Food effects on yolk androgen level	Food effects on within‐clutch variation	Notes
*Larus fuscus*	Verboven et al. (2003)	T concentrations and total amounts	Supplementation	Negative trend	Not significant	
		DHT concentrations and total amounts	Supplementation	Negative	Not significant	
		A4 concentrations and total amounts	Supplementation	Negative	Marginal insignificant	
*Taeniopygia guttata*	Rutstein et al. (2005)	T and DHT concentrations	LQ V.S. HQ in protein content	No effects	Not significant	
	Sandell et al. (2007)	T and DHT concentrations	LQ V.S. HQ in protein content Food treatment stopped before yolk formation	No effects	Decreased in LQ group; no pattern in HQ group	*Stopped before yolk formation*
*Serinus canaria*	Vergauwen et al. (2012)	T concentrations and total amounts	LQ (20 g/week) V.S. HQ (ad lib. + supplementation)	No effects	Higher increasing slope in HQ group	
		A4 concentrations and total amounts	LQ (20 g/week) V.S. HQ (ad lib. + supplementation)	No effects	Not significant	
*Rissa tridactyla*	Benowitz‐Fredericks et al. (2013)	T and A4 concentrations	Supplementation	No effects	Not significant	Multimodel inferences
*Parus major*	Ruuskanen et al. (in press)	T and A4 concentrations	Supplementation	No effects	Not significant	
*Parus major*	Giordano et al. (2014)	T and A4 concentrations	Supplementation	No effects	Not applicable	Only 4th eggs
*Larus fuscus*	Verboven et al. (2010)	T concentrations	Supplementation	No effects	Not applicable	Only 2nd eggs
*Rissa tridactyla*	Gasparini et al. (2007)	A4 concentrations and total amounts	Supplementation	Negative, but only in replacement clutches	Not applicable	Only 2nd eggs

The negative results of the field experiments have to be taken cautiously. These applied only food supplementation, but no experimental food restriction. This is understandable because of the practical difficulty to execute proper food restriction in the wild. Furthermore, the variations in natural food conditions are difficult to assess and to control for. Especially when natural food abundance is high, food supplementation may have been ineffective (Ruffino et al. 2014). Therefore we conducted a different approach in our study, using a wild species in captivity with reduction of food in both quantity and quality (see below).

In addition to androgens, egg yolks of avian species also contain other steroids, including the biological active hormones progesterone and corticosterone. Progesterone is present in high amounts, but its function on offspring development is completely unclear (von Engelhardt and Groothuis 2011). The concentration of yolk corticosterone is highly debated as its measurement is fraught with difficulty and again its effect on offspring not known (Rettenbacher et al. 2009). More interesting for our study is the presence of thyroid hormones (3,5,3′‐triiodothyronine, T3 and L‐thyroxine, T4; Prati et al. 1992), known to have effect on offspring development, but they were never studied in the context of adaptive maternal deposition and hatching asynchrony. Unlike sex steroids that are produced in the follicle wall close to the ovum, thyroid hormones are produced in the thyroid glands (McNabb et al. 1998; McNabb 2007). Therefore, thyroid hormones must be transported to the follicles via blood circulation (McNabb 2007; Groothuis and Schwabl 2008). Moreover, compared with gonadal steroids, which are produced from cholesterol that is present in much larger concentrations than its derived hormones, the production of thyroid hormones might be more costly. This is because it requires a critical constituent: iodine, whose availability largely depends on the diet and the environment (Fisher 1996). In humans, iodine deficiency can cause thyroid disorders (Zimmermann and Boelaert 2015) and even mild iodine deficiency during pregnancy can lead to hypothyroxinemia and consequently mal‐development of the foetal nervous system (Trumpff et al. 2013). Therefore, iodine availability might generate a trade‐off in the mother between allocating the hormone to self or to her eggs.

As egg mass is linked to chick quality (Krist 2011), it seems also obvious that mothers are faced with a clear trade‐off between allocating nutrients to self or the egg. In addition, mothers may also differentially allocate nutrients to the different eggs of the laying sequence in order to facilitate either rearing the complete brood or brood reduction. However, food supplementation studies in birds gave mixed results with respect to egg or yolk mass, even for those that manipulated food during egg‐laying, although these latter studies are scarce (for reviews see Nilsson and Svensson 1993; Williams 1996).

Although previous studies have attempted to look at multiple egg substances and search for potential concerting or compensating relationships (Groothuis et al. 2006a; Vallarino et al. 2012; Postma et al. 2014), the difference among various categories of egg components has not yet been addressed in relation to food condition. In this study, we used Rock Pigeons (*Columba livia*) as a model species. Rock Pigeons typically lay two eggs as a clutch and the second egg contains much higher testosterone levels than the first egg when fed ad libitum (Goerlich et al. 2009). In homing pigeons, as well as in our rock pigeon colony, the second egg is laid on average 44 h after the first egg (Levi 1998; Goerlich et al. 2010), and the two chicks usually have 24–36 h difference in hatching time (Johnston and Janiga 1995; B.‐Y. Hsu, pers. observ.) leading to a clear disadvantage of the second hatched chick. This clear case of hatching asynchrony makes the rock pigeon an excellent study species for our study. We put breeding pairs in either food ad‐libitum condition or food restricted condition while in addition we manipulated the quality of the food (see Materials and Methods). This treatment was continued until we collected their first clutch of eggs. We expected that in the food‐restricted condition, egg and yolk mass will be reduced compared with eggs laid in the food ad‐libitum condition. Based on the above the overall levels of yolk androgens may be higher in good food conditions where chicks can better bear the costs of testosterone as this egg hormone is known to suppress immune responses in the chicks (Andersson et al. 2004; Müller et al. 2005; Groothuis et al. 2006b; reviewed in Groothuis et al. 2005; Gil 2008; von Engelhardt and Groothuis 2011). We expect to see an increase of the androgen levels over the laying sequence in good food conditions in order to facilitate the rearing of the full brood and a much flatter or decreasing pattern under poor food conditions. For thyroid hormones, a newly published parallel study in great tits (*Parus major*), having much larger clutches and less clear hatching asynchrony pattern, did not find any change induced by food supplementation (Ruuskanen et al. in press). We nevertheless expect lower overall levels in eggs laid under the poor food condition because of reduced iodine availability and simultaneous iodine supplementation in the good food condition (see Materials and Methods and Appendix S1). Also, previous studies in different avian species found consistent evidence that plasma T3 levels decreased during food restriction or complete fasting (Decuypere et al. 2005; Ring dove, *Streptopelia risoria*, Lea et al. 1992; Rock pigeon, *C. livia*, Hohtola et al. 1994; Prakash et al. 1998; Japanese quail, *Coturnix japonica*, Hohtola et al. 1994; Domestic chicken, *Gallus domesticus*, Darras et al. 1995). Assuming this is also the case for egg‐laying females, yolk thyroid hormones levels are expected to be lower under poor food conditions. We did not have a clear expectation about the pattern of thyroid hormone concentrations over the laying sequence as the prior knowledge is too scarce. However, assuming T3 speeds up growth, one might expect that the second egg contains higher concentrations of this hormone only under good food conditions to compensate the last‐hatched chick when rearing the full brood is possible.

## Materials and Methods

### Animals and housing conditions

Rock pigeons (*C. livia*) are the wild ancestor of racing, show and ornamental pigeons. The species is monogamous, with a modal clutch size of two eggs. We used birds from our breeding colony, housed in a large aviary (45 m long × 9.6 m wide × 3.75 m high) at the outdoor animal facility of the Centre for Life Sciences, University of Groningen. These pigeons were descendants outbred from wild‐caught individuals. No artificial selection based on any trait had occurred and all aspects of their morphology, including body size and plumage pattern, were all consistent with the wild type (Johnston and Janiga 1995). For this study, on April 2, 2012, 20 pairs of adult pigeons (age: 2–9 years) with known pair‐bonding relationship were moved to 10 identical smaller aviaries (4.01 m long × 1.67 m wide × 2.2 m high) at the same animal facility. Two pairs of pigeons were housed in each aviary, in which two nest‐boxes with a nest‐bowl and nesting materials were provided. All pigeons were allowed to acclimatize to the new aviaries for 1 week before the experiment started. During this initial week they were provided with constantly available water and ad‐libitum standard pigeon food (KASPAR™ 6721 and KASPAR™ 6712, see Appendix S1 for nutrient components).

All experimental procedures and housing conditions were approved by the animal welfare committee of University of Groningen (DEC No. 5635D).

### Experimental procedure, food treatment and egg collection

After 1 week of acclimatization, we started the food treatment until the first clutch of eggs was completed. Each aviary was alternately assigned to either good or poor food condition. For the good food condition, pigeons received ad‐libitum seed mixture plus nutrient supplementation (ad‐libitum pigeon pellet KASPAR™ P40 and a spoon of vitamin powder, Supralith™/day). For the poor food condition, pigeons received 33 g “chicken” grain mixture per pair/day. According to our previous measurements (unpublished data), 33 g was the average food consumption by a pair of rock pigeons/day under standard food conditions when not reproducing. Breeding females demand more energy and nutrients during egg production to meet the minimal requirement of breeding. Indeed, a pilot study showed that further restriction of food to 28 g grain mixture per pair/day failed to successfully induce any egg‐laying. Further details on protein and fat content and supplementation of iodine, vitamins and other minerals in the good food condition, and restriction of both quantity and quality in the poor food condition are presented in the Appendix S1.

On May 17, we opened nest‐boxes of the poor food condition group to induce nest‐building and egg‐laying. Because we expected that the pigeons in the good food condition group would start egg‐laying earlier than those in the poor food condition group, we opened nest‐boxes of the good food condition group a few days later, on May 21. All nest‐boxes were checked every morning to ensure all eggs were collected within 24 h after being laid. We successfully collected 6 and 8 full clutches of eggs in the good and poor food condition, respectively, from May 22 to June 6. Collected eggs were always replaced by dummy eggs. At the day of collection we measured the length and width of the egg to the nearest 0.01 mm with a digital calliper and egg weight to the nearest 1 g with a digital scale. Egg size was estimated by the equation:

$$V=0.51\;LW^{2},$$

where *V*,* L*,* W* represent the volume, length, and width of an egg, respectively; 0.51 is a volume coefficient (Hoyt 1979; Johnston and Janiga 1995). All collected eggs were then immediately frozen at −20°C until hormone extraction and assay.

### Hormone extraction and assays

We used radioimmunoassay to quantify the concentrations of four hormones: testosterone (T), androstenedione (A4), triiodothyronine (T3), and thyroxine (T4), in pigeon egg yolks. Before extracting hormones, we thawed the frozen eggs for a few minutes to remove egg shells, and the yolk and albumin were carefully separated. Every yolk was then weighed on an analytical balance (accuracy 0.001 g) in a sterile 50 mL centrifuge tube and 2600 μL MilliQ water was added. This yolk/MilliQ water mixture of each egg was then stored in −20°C until we performed hormone extraction and radioimmunoassay.

#### Androgens (T and A4)

To extract T and A4, 225–287 mg of yolk/MilliQ water mixture (1 + 1) was weighed (accuracy 0.001 g), 300 μL of MilliQ water and 50 μL of ^3^H‐labeled testosterone were added to trace the recovery of extracted hormones during the extraction procedure. This solution was incubated for 15 min at 37°C before being extracted in 2 mL of diëthylether/petroleumbenzin (DEE/PB, 70/30 v/v) by vortexing for 60 sec. Extracted samples were centrifuged at 672 g for 3 min (4°C) to separate the ether phase. The samples were snap‐frozen and the ether/hormone phase was decanted into a fresh 5 mL tube. The extraction procedure was repeated twice with an additional 2 mL of DEE/PB, vortexed for 30 and 15 sec respectively. Next, the extracts were dried under nitrogen. Hormone extracts were rinsed in 2 mL of 70% methanol to precipitate any lipids and stored overnight at −20°C. Subsequently, the tubes were centrifuged at 2000 rpm for 5 min (4°C), decanted into a fresh 5 mL tube, redried under nitrogen and stored at −20°C.

Prior to the assay, extracts were dissolved in, respectively, 250 μL (1st egg yolk) and 500 μL (2nd egg yolk) of phosphate‐buffered‐saline with gelatin. From this solution, respectively, 50 μL (1st egg yolk) and 100 μL (2nd egg yolk) was mixed with scintillation cocktail (Ultima Gold; Perkin Elmer, Groningen, the Netherlands) and radioactivity counted on a liquid scintillation counter. Subsequently, 25 μL of each sample was used for T determination using a kit purchased from Orion Diagnostica (“Spectria 68628”; Espoo, Finland, cross reactivity to A4 and 5α‐DHT was 1.7% and 2.6, respectively, all others <0.31%). For A4 determination 50 μL of sample (×21 dilution) using a kit purchased from Beckman Coulter GmbH (“DSL‐3800”; Sinsheim, Germany, the cross reactivity to A4 and T was both <0.1%). Standards were prepared using dilution series from pre‐prepared stock and ranged from 0.08–20 ng/mL for T and 0.16–20 ng/mL for A4. Own dilution curves ran parallel with the standards. Recoveries averaged 85% (SD 4.3%). “Pools” of yolk were used as external controls and intra‐assay CV for T was 2.6%, and intra‐assay for A4 was 5.8%.

#### Thyroid hormones (T3 and T4)

The concentrations of T4 and T3 concentrations were measured by radioimmunoassay (Darras et al. 1990) following extraction of the tissues as described in detail earlier (Reyns et al. 2002). In short, 600 μL yolk/MilliQ water mixture was homogenized in a methanol volume three times the mixture's weight. As individual internal recovery tracers, 1500–2000 cpm of outer ring labeled ^[131I]^T3 and ^[125I]^T4 were added. A volume of chloroform, twice the volume of methanol, was added. After centrifugation (15 min, 1900 *g*), the pellet was re‐extracted in a mixture of chloroform and methanol (2:1). Back‐extraction into an aqueous phase (0.05% CaCl_2_) was followed by a re‐extraction with a mixture of chloroform:methanol:0.05% CaCl_2_ (3:49:48) and this phase was further purified on Bio‐Rad AG 1‐X2 resin columns. The iodothyronines were eluted with 70% acetic acid, evaporated to dryness and resuspended in RIA buffer. Typical recoveries of extracted thyroid hormones ranged from 55 to 75% for T3 and from 40 to 60% for T4. The T3 RIA had a detection limit of 2 fmol and an intra‐assay variability of 2.2%. The T4 RIA had a detection limit of 5 fmol and an intra‐assay variability of 2.8%. For the T3 RIA cross‐reactivity with T4 was 0.1–0.5%, whereas for the T4 RIA cross‐reactivity with T3 was 3.5%. All samples were measured within a single assay.

### Statistical analysis

We used linear mixed‐effects models (r package *lme4*, Bates et al. 2014) to test the effects of food treatment on egg mass, egg size, yolk mass, and hormone levels in egg yolks. The identity of the nest where eggs were laid was included as a random factor. Laying order, food treatment and their interaction were included as fixed factors. First we tested whether these fixed factors had main effects on egg characteristics and yolk hormones and hence the model did not include any interaction term. Secondly, we specially tested the interaction between laying order and food in each statistical model, as this interaction represented the change of the within‐clutch pattern. We additionally tested the association between yolk mass and the concentrations of the four measured yolk hormones in separate models, because yolk mass was significantly affected by our food treatment (see Results). All model residuals were visually inspected to check for homogeneity and normality. To check for collinearity, we calculated VIF values by the function *vif.mer* (HLP/Jaeger lab blog, 2011). All VIF values were lower than 2 and no considerable collinearity occurred. All *P* values were derived by log‐likelihood ratio tests. Mean ± SD are presented unless mentioned otherwise. All statistics were conducted with r version 3.0.2 (R core team 2013). Alpha was set at 0.05. Because of the relative small sample size in each treatment, *P*‐values between 0.1 and 0.05 are described as a nonsignificant trend.

## Results

### Laying date

The interval from breeding induction (nest‐box opening) to the laying date of the first egg was significantly longer for pigeons in the poor food condition (median: 10.5 days) than pigeons in the good food condition (median: 6.5 days; Mann**–**Whitney *U* test, *P* = 0.023), suggesting that our food treatment successfully created difference in female physiological condition for breeding. Since we intentionally delayed opening the next‐boxes for the good‐food group (see Materials and Methods), there was no significant difference in laying date of the first eggs between the different groups (Mann**–**Whitney *U* test, *P* = 0.7433).

### Egg mass and yolk mass

**Figure 1:**
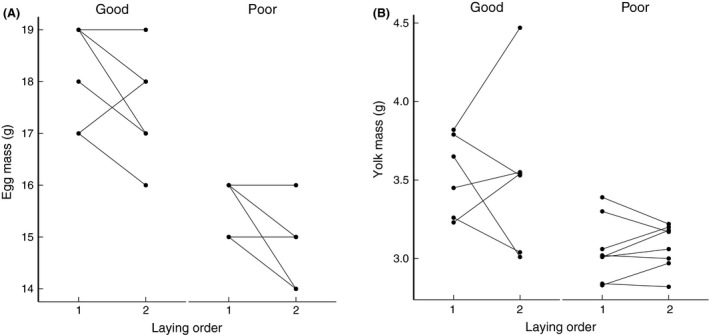
In the poor‐food condition, pigeons laid eggs with lower mass (A) and smaller yolk (B). Good: good‐food condition; Poor: poor‐food condition.

Our food treatment had significant effects on egg mass and yolk mass, without interacting with laying order. The eggs laid by pigeons in the poor food condition were significantly lighter (*P* < 0.0001, Table 2, Fig. 1A) and with lighter yolks (*P* = 0.0024, Table 2, Fig. 1B). Because of the strong correlation between egg mass and yolk mass (Pearson's correlation, *r* = 0.6, *P* = 0.0007), we also extracted the residuals of yolk mass against egg mass from a linear model and analysed these with the same mixed‐effects model. This model showed that there was no longer a significant effect of food treatment (*P* = 0.4775), suggesting that yolk mass was not disproportionately affected by our food treatment.

**Table 2:** Results of linear mixed‐effects models on egg parameters. All statistics about main effects were from the models leaving out all interaction effects

	Estimates	SE	*t*	*P* a
Egg mass				
Laying order (2nd egg)	−0.7857	0.2143	−3.670	0.0016
Food (poor)	−2.6458	0.3755	−7.050	<0.0001
Laying order – Food	−0.2083	0.4467	−0.470	0.6160
Yolk mass				
Laying order (2nd egg)	0.0071	0.0802	0.089	0.9264
Food (poor)	−0.4608	0.1376	−3.348	0.0024
Laying order – Food	0.0300	0.1685	0.178	0.8476
Residuals of yolk mass against egg mass				
Laying order (2nd egg)	0.1158	0.0706	1.639	0.1049
Food (poor)	−0.0951	0.1433	−0.664	0.4775
Laying order – Food	0.0588	0.1476	0.398	0.6679

T, testosterone; A4, androstenedione.

*P* values were derived from log‐likelihood ratio tests.

Laying order also significantly affected egg mass. The second eggs of a clutch were significantly lighter than the first eggs (*P* = 0.0016; Table 2, Fig. 1A). There was no significant interaction effects between laying order and food treatment on egg mass (*P* = 0.616, Table 2, Fig. 1A).

Interestingly, despite the correlation between egg mass and yolk mass, the yolk mass of two eggs within a clutch did not show significant differences (*P* = 0.1049, Table 2, Fig. 1B). This indicates that the within‐clutch variation in egg mass is mainly caused by the difference in the amount of albumen instead of yolk.

### Yolk androgens

**Figure 2:**
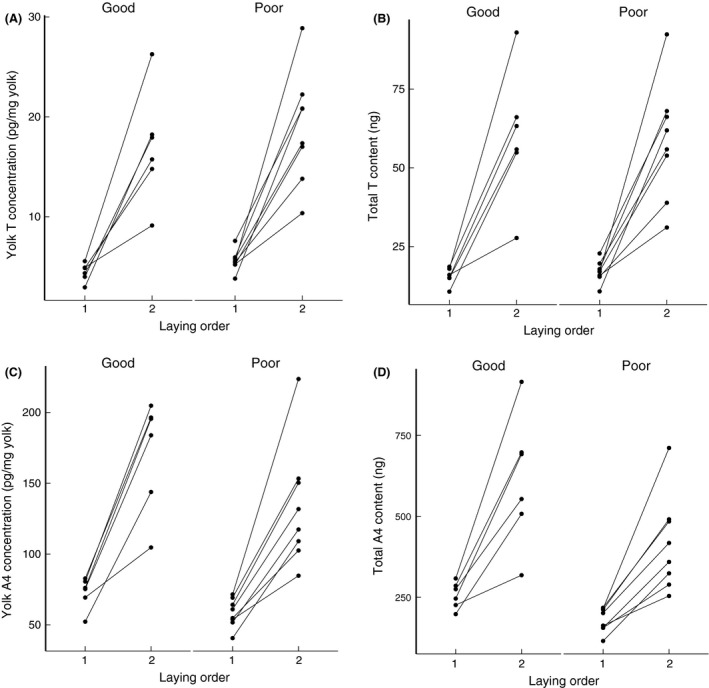
The second eggs contained higher T and A4 in the yolks, but food treatments did not affect yolk T (A, B). Yolk A4 concentration (C) in the eggs laid under the poor‐food condition was almost significantly lowered and the total A4 content (D) was significantly reduced. T: testosterone; A4: androstenedione. Good: good‐food condition; Poor: poor‐food condition.

T and A4 concentrations and their total amount per egg showed a very strong increase over the laying sequence (T concentration, *P* < 0.0001; A4 concentration, *P* < 0.0001; Table 3, Fig. 2A and C. T amount, *P* < 0.0001. A4 amount, *P* < 0.0001, Table 3, Fig. 2B and D). However, food conditions did not affect T levels, but almost significantly decreased the concentrations of A4 and significantly decreased the total content of A4 in the poor food condition (Table 3, Fig. 2). Nonetheless, the within‐clutch pattern of yolk androgens was not changed due to different food conditions, as the interaction between laying order and food treatment was not significant (*P* > 0.11, Table 3.)

**Table 3:** Results of linear mixed‐effects models on yolk androgen depositions. All statistics about main effects were from the models leaving out all interaction effects

	Estimates	SE	*t*	*P* a
Yolk T concentration				
Laying order (2nd egg)	13.013	1.437	9.054	<0.0001
Food (poor)	1.518	1.576	0.963	0.3073
Laying order – Food	0.765	3.015	0.254	0.7842
Total amount of T				
Laying order (2nd egg)	42.797	4.952	8.643	<0.0001
Food (poor)	−0.045	5.624	−0.008	0.9931
Laying order – Food	−3.100	10.376	−0.299	0.7474
Yolk A4 concentration				
Laying order (2nd egg)	85.627	9.459	9.053	<0.0001
Food (poor)	−25.892	13.414	−1.930	0.0517
Laying order – Food	−23.040	18.750	−1.229	0.1976
Total amount of A4				
Laying order (2nd egg)	288.700	41.010	7.039	<0.0001
Food (poor)	−137.400	54.890	−2.503	0.0153
Laying order – Food	−120.730	78.900	−1.530	0.1142

T, Testosterone; A4, androstenedione.

a *P* values were derived from log‐likelihood ratio tests.

**Figure 3:**
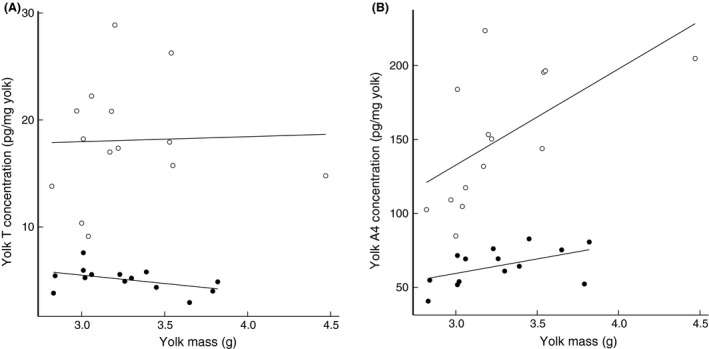
Yolk mass was not correlated with T concentration (A) but showed a significant positive correlation with A4 concentration (B) in egg yolks. Filled dots: 1st eggs of clutches; open dots: 2nd eggs of clutches.

Regardless of the position of the egg in the laying sequence, we found that yolk T concentration did not show a correlation with yolk mass (*P* = 0.910, Fig. 3A). However, yolk A4 concentration showed a significant positive relationship with yolk mass (*P* = 0.004, Fig. 3B).

### Yolk thyroid hormones

Overall, the average concentration of yolk T3 in pigeon eggs was 1.10 ± 0.21 ng/g yolk and the average concentration of yolk T4 was 3.06 ± 0.99 ng/g yolk.

**Figure 4:**
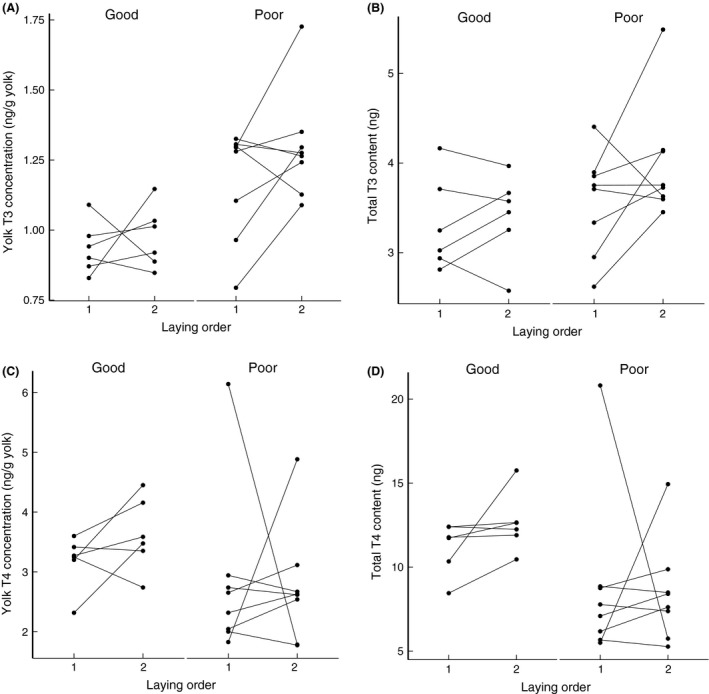
Food condition before and during egg‐laying significantly affected T3 and T4 in the egg yolks. Eggs laid under the poor food condition contained significantly higher yolk T3 concentration (A) and non‐significantly higher total T3 content (B). In contrast, yolk T4 concentration in the eggs laid under the poor food condition tended to be lower but not significantly (C), while the total T4 content was significantly lower (D).

The concentration and total amount of T3, but not of T4, showed a nonsignificant increase over the laying order (Table 4, Fig. 4). T3 concentrations were significantly higher in the eggs laid under the poor food condition and the total T3 content also tended to be higher although this did not reach statistical significance (T3 concentration, *P* = 0.0006. T3 amount, *P* = 0.0977, Table 4, Fig. 4A and B). In contrast, yolk T4 concentrations showed a nonsignificant trend to be lower in poor food conditions, and this was significant for the total T4 content (T4 concentration, *P* = 0.090. T4 amount, *P* = 0.010; Table 4, Fig. 4C and D). In none of these cases there was a significant interaction between the laying order and food condition.

**Table 4:** Results of linear mixed‐effects models on depositions of thyroid hormones in egg yolks. All statistics about main effects were from the models leaving out all interaction effects

	Estimates	SE	*t*	*P* a
Yolk T3 concentration				
Laying order (2nd egg)	0.088	0.052	1.703	0.0931
Food (poor)	0.279	0.071	3.945	0.0006
Laying order – Food	0.086	0.106	0.809	0.3885
Total amount of T3				
Laying order (2nd egg)	0.285	0.167	1.704	0.0929
Food (poor)	0.412	0.256	1.611	0.0977
Laying order – Food	0.326	0.339	0.962	0.3077
Yolk T4 concentration				
Laying order (2nd egg)	0.147	0.367	0.400	0.6723
Food (poor)	−0.609	0.371	−1.642	0.0903
Laying order – Food	−0.534	0.749	−0.713	0.4437
Total amount of T4				
Laying order (2nd egg)	0.403	1.246	0.323	0.7325
Food (poor)	−3.250	1.259	−2.581	0.0101
Laying order – Food	−1.788	2.544	−0.703	0.4500

T3, Triiodothyronine; T4, thyroxine.

a *P* values were derived from log‐likelihood ratio test.

**Figure 5:**
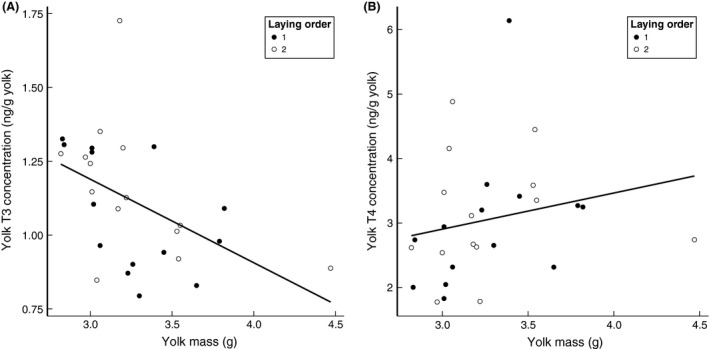
Yolk mass was negatively correlated with T3 concentration in the egg yolks (A) but not with yolk T4 concentration (B). Filled dots: 1st eggs of a clutch; open dots: 2nd eggs of a clutch.

Yolk mass showed a significantly negative correlation with T3 concentrations (*P* = 0.017; Fig. 5A), but did not have a significant correlation with T4 concentrations (*P* = 0.267; Fig. 5B).

## Discussion

In this study, we investigated the effect of food restriction on three aspects of avian egg quality: macro‐nutrients, thyroid hormone concentrations, and androgens. In accordance with our expectation, the eggs laid under the poor food condition were significantly lighter in egg and yolk mass. The results are consistent with previous studies, suggesting that these egg characteristics are to a certain extent constrained by maternal nutrient and energy ingestion prior to or during egg‐laying (Ruuskanen et al. in press; Williams 1996; De Neve et al. 2004; Ardia et al. 2006; Karell et al. 2008; Barrionuevo et al. 2014). Food restriction did not disproportionally affect the yolk mass after controlling for egg mass, probably because during egg formation, yolk is formed before albumen and eggshell are wrapped up, and yolk mass is probably the determinant of albumen mass instead of the other way around (Williams 2012).

The effect of food treatment seems within the ecological relevant range. In our colony, the egg mass laid by pigeons fed with standard ad libitum food varies from 13 to 20 g (*n* = 475, mean ± SD: 16.52 ± 1.42 g, unpublished data). Under these conditions occasionally some very small eggs of only 6–10 g are found and these usually do not have yolks. In this experiment, the smallest eggs were 14 g (Fig. 1A) and all eggs contained yolks, so that the effect on egg mass was well within the natural range.

The results on the yolk hormones were in accordance with our hypothesis, in so far that T levels did not change under poor food condition as T production would not be costly. The lack of a statistical significant effect in testosterone is unlikely to be due to our sample size as the effect size of the treatment on clutch average T concentrations was small and a power analysis revealed that only with a sample size of 60 in each group the result would reach statistical significance. Such a sample size of total 120 pairs is very large and the effect size so small that it may not have any biological meaning. This is consistent with the majority of previous studies as we reviewed in Table 1. Since yolk T concentrations and amounts appeared to be independent of yolk mass, there is probably no nutrient constraint on the deposition of yolk T. Contrary with our expectation, we did not find that the within‐clutch yolk androgen variation was affected by food treatment. There are several potential explanations for this: First, pigeon mothers might be unable to predict the food condition during chick‐rearing based on the food condition during egg‐laying, making such an adjustment nonfunctional. Second, the mothers are unable to regulate deposition of T in their eggs. Third, adjusting the T deposition according to food conditions might not be adaptive. Since incubation (18 days) and chick rearing (about 4 weeks) in this species is relatively long, the first hypothesis might be correct, but so far measuring food predictability and the perception of food predictability by the birds is still very challenging. The second hypothesis seems not likely as many studies in many bird species have shown effects of environmental factors on maternal T deposition in the egg (for a review see Groothuis et al. 2005; Gil 2008; von Engelhardt and Groothuis 2011). The third hypothesis is not unlikely. There is evidence that in female birds, T allocation to the egg is regulated independently from its allocation to the female's circulation (Groothuis and Schwabl 2008; Okuliarová et al. 2010) and that food conditions can affect the activity of the HPG axis (Davies and Deviche 2014). So perhaps our food condition affected female T levels whereas the mothers kept the concentration of this hormone in the egg unchanged. However, since we were afraid to interrupt egg‐laying we did not check for female T concentrations.

The lack of the effect of food on T deposition does not necessarily rule out a role for this hormone in the switch from rearing the full brood under good conditions to brood reduction under poor food condition. There is evidence that yolk T carries costs for the chick too, such as on the immune system and metabolic rate (Andersson et al. 2004; Müller et al. 2005; Groothuis et al. 2006b; Tobler et al. 2007; Nilsson et al. 2011; Ruuskanen et al. 2013). Perhaps only chicks in good food condition can withstand these costs relatively well so that elevated yolk T concentrations may be beneficial for chicks in good, but detrimental in poor food conditions leading in the latter case to brood reduction. Then keeping yolk T deposition unchanged would be the most evolutionarily parsimonious way to achieve brood size optimization under different food conditions. However, although similar ideas have been proposed in the literature (e.g. Royle et al. 2001; Groothuis et al. 2005), so far the direct experimental data are still lacking.

Our data suggest that yolk A4 is more sensitive to food treatment than T since the food treatment had a highly significant positive affect on total A4 (Table 2D; Fig. 3). Literature suggests that yolk A4 is more sensitive to the environmental context than yolk T (Tschirren et al. 2009). According to our data, yolk A4 concentration also showed a positive correlation with yolk mass (Fig. 3B). This probably implies an increasing A4 deposition rate with yolk formation, possibly because larger follicles have more hormone‐producing cells or because the lipophilic A4 deposition is “hitch‐hiking” on yolk deposition. However, we did not see a similar positive correlation for yolk T (Fig. 3A), implying that A4 is deposited via a somewhat different mechanism from T unless A4 is converted to T by maternal enzymes that are deposited in the yolk too.

### Yolk thyroid hormone levels and their regulation

The concentrations of yolk thyroid hormones (THs) in pigeon eggs are comparable with all currently known data in other species (chickens and quails, T3: 1.5–2.5 ng/g yolk, T4: 4–6 ng/g yolk, McNabb and Wilson 1997; great tits, T3: 0.12–0.59 ng/g yolk, T4: 1.5–6.6 ng/g yolk, Ruuskanen et al. in press). Concentrations of T3 were highly significantly increased under poor conditions (Table 4, Fig. 4). To our knowledge, our study and the other newly published complementary study (Ruuskanen et al. in press) are the only two that experimentally examining the effects of food availability on avian yolk THs levels so far. Ruuskanen et al. (in press) applied food supplementation in a wild great tit population while we did food restriction in a captive wild pigeon colony. Our results are in agreement for the changes in egg mass, yolk mass, and the levels of two yolk androgens. However, Ruuskanen et al. (in press) did not found a significant change in yolk TH levels like in our study. It is likely that our food restriction design in captivity created a stronger difference in iodine availability between groups than in the great tit field study in which food availability was not completely under the experimenter's control.

Unlike sex steroids, which are produced close to the ovum in the follicle wall, THs are secreted in the thyroid glands. Therefore, the most likely pathway for egg yolks to accumulate THs is via the blood circulation, either by active transport or passive diffusion (McNabb and Wilson 1997). Previous studies consistently showed lowering of plasma T3 under complete fasting or partial food restriction (Lea et al. 1992; Darras et al. 1995), even in the same species as in our study (Hohtola et al. 1994; Prakash et al. 1998). Since we found elevated instead of lower yolk T3 levels under food restriction, passive diffusion in which yolk concentrations mirror maternal circulating concentrations is unlikely. Although we did not measure the plasma T3 levels in our pigeons in order not to interrupt their breeding, according to the literature in the same species and other species (Hohtola et al. 1994; Prakash et al. 1998), our results suggest a regulation of T3 into egg yolks independent from the blood circulation. Apolipoprotein D, transthyretin, and even vitellogenin and very low density lipoprotein are all possible candidates for transporting THs into egg yolks (McNabb and Wilson 1997). Because transthyretin is the main TH distributor in birds (Schreiber 2002; Richardson 2009) and avian transthyretin has higher affinity with T3 than T4 (Chang et al. 1999), the expression of transthyretin along with other TH transporters (e.g. MCT8, OATP1C1, etc.) in ovarian follicles would be worth of examination under different food conditions.

Alternatively, differential enzymatic activity in ovarian or follicular tissue between the food conditions might be responsible for our results. Unlike T4, which is mainly secreted by thyroid glands, most of the circulating T3 is converted from T4 via the activity of deiodinases in peripheral tissues (Gereben et al. 2008). Therefore, T3 levels in a local tissue can be regulated by deiodinases independently of the plasma THs levels (Gereben et al. 2008). If the activity of deiodinases in the ovarian follicles or nearby tissues, or even in the yolk itself, during food restriction favors the conversion of T4 to T3, we would see the increased T3 accumulation accompanied with a reduction in T4 concentration in egg yolks for which we found a nonsignificant trend. The situation could be more complex, as our data also indicate a larger amount of decrease in yolk T4 than the amount of increase in T3 (Fig. 4). Also, considering the small sample size and the large variation in yolk T3 and T4 under the poor food condition, further studies will be valuable in shedding more light on this part.

T3 and T4 in the egg yolks also showed a different relationship with yolk mass. We found a significant negative correlation between yolk mass and yolk T3 concentration, but not T4. This suggests that the deposition of T3 is relatively constant, and becomes more diluted with increased deposition of yolk. The negative correlation also suggests that the higher T3 concentrations in the egg yolks under poor food condition can be explained as an indirect effect due to the reduced yolk mass. Indeed, the total amount of T3 did not change significantly due to the treatment. Interestingly, the reverse was true for T4, since not its concentration but the total amount per yolk changed significantly with treatment while there was no correlation between T4 concentration and yolk mass. Thus a decrease in yolk mass due to poor food was accompanied by a similar decrease in T4 amounts. Perhaps smaller yolks are less vascularized, leading to both less migration of T4 and vitelline from the circulation to the yolk. Or, T4 hitch‐hikes on yolk deposition so that less deposition of yolk precursors also leads to less deposition of T4. Then, in the follicle or ovum, T4 is converted to T3, independently regulated from T4 levels.

Interestingly, both the precursors A4 and T4 decreased under food restriction, especially in their total amount per egg yolk, in contrast with their biological active counterparts T and T3, respectively, despite that the first is produced in the follicle wall and the other in a distant gland. This difference in deposition mechanism suggests that it is not so much their pattern of deposition but their conversion in the yolk that showed a similar effect in response to the food treatment.

### Yolk thyroid hormone levels and their potential function

Maternally derived thyroid hormones have been proven to be crucial in embryonic brain development (mammals: Morreale de Escobar et al. 2004a, 2004b; chicken: Flamant and Samarut 1998; Van Herck et al. 2013). In birds, yolk THs are very likely to play important roles in embryonic development because the nuclear receptors, membrane transporters, and the deiodinases which can locally regulate THs availability are all demonstrated to be present in avian embryos far before the embryonic thyroid function (Flamant and Samarut 1998; Darras et al. 2011; Van Herck et al. 2012). In Japanese Quails (*C. japonica*), Wilson and McNabb (1997) showed that T4‐treated females laid eggs containing both higher levels of T3 and T4 in the yolks, and the embryos in the eggs had enhanced development of pelvic cartilage. This suggests that higher TH levels in egg yolks may enhance the development at TH‐responsive tissues. If this is also the case in Rock Pigeons, the higher levels of THs in the eggs laid under the poor‐food condition may represent a maternal effect of stimulating embryo development. Unfortunately, effects of T3 or T4 have not yet been experimentally tested by *in ovo* injections. Although the data about the effects of elevated prenatal exposure to THs in bird species are scarce, studies in lab rodents and humans have shown crucial effects of prenatal THs on normal brain development. Whether in birds the elevated TH exposure might eventually lead to enhanced cognitive ability, as found in mammalian studies, or other phenotypic effects that might enhance fitness is an interesting topic for further studies. An alternative explanation is that under poor food conditions, egg‐laying mothers enhance the conversion from T4 to T3 in the egg as each conversion delivers an iodine that might have been limiting in the poor food condition. However, this is not consistent with the finding that the concentration of T3 decreases in the blood circulation of the mother itself under food restriction.

In conclusion, we found that female pigeons may regulate different components of egg quality in different manners when facing poor food condition. This suggests the maternal effects mediated by these components are under different constraints and maternal regulatory pathways. Our results also provide supporting evidence for the possibility that deposition of hormones in the egg can be independent from that in the mother's circulation, providing her with much more flexible tool for maternal effects than often assumed. The results also open the possibility that mothers not only deposit hormones in their eggs, but also make use of the relevant enzymes in the ovary to convert hormone precursors into their biological much more active metabolites or deposit these enzymes in the yolk itself. Furthermore, the results indicate that to understand environmentally induced maternal effects, studying only one pathway for such effects may be misleading. Finally, the results indicate that thyroid hormones seem to be an intriguing case of a costly maternal signal that warrants much more study.

## Data Accessibility

All data are included in the manuscript and supporting information.

## Conflict of Interest

None declared.

## Supporting information


**Appendix S1.** Macronutrient components of grain mixtures in food treatment.

**Appendix S2.** Data of food and egg quality in pigeons.
