# Supplementary material for: Maternal adjustment or constraint: differential effects of food availability on maternal deposition of macro‐nutrients, steroids and thyroid hormones in rock pigeon eggs
Source: Ecol Evol. 2016 Jan 8;6(2):397–411. doi: 10.1002/ece3.1845 (PMC4729257; doi:10.1002/ece3.1845)
Supplement: Supplementary file 1 — Appendix S1. Macronutrient components of grain mixtures in food treatment. [file ECE3-6-397-s001.docx]

**Appendix A.**

Macronutrient components of grain mixtures in food treatment.

| Name of grain mixture | Tortelduivenvoer (KASPER^TM^ 6721) | Sierduivenvoer (KASPER^TM^ 6712) | Duivenkorrel^a^ (KASPER^TM^ P40) | Gemengd graan met broken mais |
| --- | --- | --- | --- | --- |
| Protein | 11.90% | 13.30% | 15.60% | 10.10% |
| Fat | 5.20% | 3.40% | 2.80% | 2.40% |
| Cellulose | 6% | 4.40% | 2.10% | 2.80% |
| Ash | 2.40% | 2.40% | 4% | 1.60% |
| Standard food | *ad lib.* | *ad lib.* |  |  |
| Good food^b^ | *ad lib.* | *ad lib.* | *ad lib.* |  |
| Poor food |  |  |  | 33g /pair-day |

^a^ also contains calcium 0.6%, potassium 0.6%, lysine 6g, sodium 0.1%, E 672 vitamin A 19000 lE/kg, vitamin E 100 lE/kg, E 671 vitamin D3 300 lE/kg, and copper 12mg/kg

^b^ also supplemented with Supralith TM supplementation powder every one or two days. Supralith contains vitamin A 9000 IE/kg, vitamin D3 3000 IE/kg, vitamin E1 8.5 mg/kg, Calcium 17%, linolz 0.5% and raw ashes 80%. Supralith also contains potassium iodide, which provides iodide 1.5 mg/100g.
